# Factors associated with ART adherence among HIV-positive adherence club members in Ekurhuleni Metropolitan Municipality, South Africa: A cross-sectional study

**DOI:** 10.1371/journal.pone.0277039

**Published:** 2022-11-01

**Authors:** Tariro Ndoro, Ntombizodwa Ndlovu, Peter Nyasulu

**Affiliations:** 1 Division of Epidemiology and Biostatistics, School of Public Health, Faculty of Health Sciences, University of the Witwatersrand, Johannesburg, South Africa; 2 Division of Epidemiology and Biostatistics, Faculty of Medicine and Health Sciences, Department of Global Health, Stellenbosch University, Cape Town, South Africa; University of Limpopo, SOUTH AFRICA

## Abstract

**Background:**

HIV is a leading cause of morbidity and mortality in South Africa that can be managed using antiretroviral therapy (ART). Adherence clubs are interventions that have been introduced to decentralize ART to improve ART adherence and provide social support for club members. However, ART adherence can be suboptimal even among adherence club members.

**Aim:**

This study aimed to determine the factors affecting ART adherence among people living with HIV/AIDS (PLWHA) attending adherence clubs in Ekurhuleni Metropolitan Municipality.

**Methods:**

A cross-sectional study was conducted. Ordinal logistic regression was used in univariable and multivariable analyses to determine factors significantly associated with adherence scores. Factors included in the final model were age, comorbidity, ART regimen and club membership duration.

**Results:**

The records of 730 participants were analysed. After adjusting for age, participants with comorbidities were half as likely to report high ART adherence scores compared to participants without comorbidities (AOR = 0.5, 95% CI: 0.3–0.8, p = 0.005). The adjusted odds of reporting high levels of adherence among participants on cART were 1.8 times those on a single tablet regimen (AOR = 1.8, 95% CI: 1.0–3.2; p = 0.033). There was a 20% reduction in the adjusted odds of reporting high ART adherence for each additional year of adherence club membership (AOR = 0.8, 95% CI: 0.8–0.9, p<0.001).

**Conclusion:**

Increasing years spent as adherence club members, single tablet ART regimens and the presence of comorbidities were all significantly associated with low ART adherence among study participants. Regular assessment of the quality of counselling sessions for ART adherence club members and questionnaires for early screening of treatment fatigue have been suggested as tools for improved adherence in ART adherence club settings.

## Background

With a total of 7.6 million people living with HIV/AIDS (PLWHA), South Africa (SA) has the world’s highest HIV prevalence [1]. In South Africa, free antiretroviral therapy was only rolled out in 2004 [2], and the current study was conducted over a decade later. Although SA has the world’s largest ART programme [3], retention in care and ART adherence remain suboptimal because of competing pressures on the healthcare system to initiate more PLWHA onto ART whilst maintaining those already in care [2]. Nonadherence among PLWHA has public health ramifications such as increased morbidity, mortality, antiretroviral resistance, and viral transmission [4].

In the current standard of care (SOC), PLWHA visit clinicians every two months to receive care (symptom screening, WHO staging, adherence counselling, and weight testing) and ART [5]. The ART adherence club intervention is a differentiated service delivery (DSD) model that relies on task shifting and decentralization of care for clinically stable patients [6,7] to reduce patient loads on clinics, allowing the prioritization of critical patients by clinicians in SOC [8,9]. Clinically stable patients are defined as adult (≥ 18 years) PLWHA who have been on the same ART regimen for at least 18 months, have high CD4 counts, undetectable viral loads (<400 copies/mL), and no or reasonably managed comorbidities [8].

In the adherence club model reported here, clinically stable PLWHA attend meetings at two or three-month intervals. These consist of: (i) distribution of pre-packaged ART and (ii) counselling sessions on HIV-related matters facilitated by lay community health workers (CHWs). People living with HIV/AIDS in adherence clubs still need to visit clinics twice a year for check-ups and SOC where their viral loads and CD4 counts are measured every six months [6,8,10]. Adherence club members are reverted to bimonthly SOC if they miss meetings, fail to collect medication, experience virological rebound or develop complications with a comorbidity, thus ensuring that they receive specialized medical attention from a clinician [6].

Few studies have investigated factors influencing ART adherence among adherence club members. In studies assessing factors affecting adherence in SOC or factors affecting viral load suppression and retention in care in adherence clubs, several determinants of ART adherence have been identified, including age [11–13], sex [13–17], marital status [13–17], highest educational attainment [12,16,18,19] and employment [14,20,21]. The presence of comorbidities [16], the specific ART regimen [16,22–25] and the duration of ART treatment [12,26,27] are some of the clinical factors that have been associated with ART adherence.

Suboptimal conditions have been increasingly observed in some adherence clubs. As the intervention has been scaled up, some adherence clubs have stopped providing health counselling ART [28,29] or club facilitators were increasingly overwhelmed by their workloads as the adherence clubs grew larger [30]. Such conditions may negatively impact the gains initially made by the introduction of adherence clubs, emphasizing the need to optimize the adherence club model and thus retain patients.

In a 2016 seroprevalence survey, Ekurhuleni Metropolitan Municipality was found to have the second-highest HIV prevalence (15.0%) of all metropolitan municipalities in South Africa [31,32]. Since adherence clubs are a key intervention for improving treatment outcomes for PLWHA [8] and Ekurhuleni is home to many PLWHA, it is important to have a good understanding of adherence club function within this context. Inefficiencies in the adherence club model, the dearth of studies investigating ART adherence as an outcome, and the high prevalence of HIV in Ekurhuleni Metropolitan Municipality provided an opportunity to study the factors affecting ART adherence in an adherence club setting. Hence, this study sought to determine factors affecting ART adherence among clinically stable HIV-positive individuals attending ART adherence clubs in Ekurhuleni Metropolitan Municipality in Gauteng province, South Africa.

## Methods

### Study setting

Adherence clubs are run at clinics with high volumes of patients on ART. The adherence clubs included in this study were all facilitated by the then Community AIDS Response (CARe), in Norwood, Johannesburg. Clinically stable PLWHA were invited to join adherence clubs by their doctors. Both adherence club facilitators and club members were counselled on the importance of maintaining the confidentiality of fellow adherence club members. Club meetings were held once every two months in the mornings to allow employed members to go to work after the meetings. Each clinic had a designated room for adherence club members to meet. After undergoing symptom screening and then collecting their antiretroviral medication, club members attended an HIV-centred group counselling session [33]. The clubs had been operating for more than a year at the time of data collection.

The details of the sampling strategy are provided in Tshuma, 2017 [33]. The primary study comprised 730 participants who were systematically sampled from 16 adherence clubs in Ekurhuleni, Gauteng Province. The number of participants per adherence club was calculated proportionately to the number of club members at each site. Participants were selected using a sampling frame until the quota for each site was reached. Two research assistants were employed to collect data. They recruited study participants before the beginning of adherence club sessions by approaching club members whose names were in the sampling frame. Club members who granted informed consent were recruited into the study. A paper-based questionnaire was then administered in English to the participants by the two research assistants.

### Study design

This was a cross-sectional study using secondary data provided by CARe. The study population comprised of clinically stable HIV-positive adults, above 18 years of age, who were members of ART adherence clubs in Ekurhuleni Metropolitan in February 2016.

### Data management

#### Data sources

Data were imported from Microsoft Excel into STATA 15.0 (StataCorp. Ltd, College Station, TX, USA) for analysis [34]. All the data from the original study were used in this study.

#### Variables

*Outcome variable*. The outcome variable for this study was self-reported ART adherence, measured using four items, on a 5-point Likert scale. The items were based on responses to the following statements: ‘adherence clubs make me take my medication at the right time’; ‘I collect my medication on appointed dates’; ‘clubs make me not miss taking my medication daily’; and ‘clubs make me adhere to treatment’. For each participant, a mean variable “adherence” was calculated using the individual Likert scores from each item/question, giving a continuous variable with values ranging from 1 to 5. The ordinal outcome variable “adherence score” was categorized into highly nonadherent (≤1.4) nonadherent (1.5–2.4), neutral (2.5–3.4), adherent (3.5–4.4) and highly adherent (≥4.5).

*Exposure variables*. The sociodemographic variables collected included sex, age, marital status, education, and employment status. Age in years was recoded into the following categories: 18–30 years, 31–50 years and >50 years. Marital status was originally collected as single, co-habiting, married, divorced, and widowed and recoded to single, married/cohabiting, and divorced/widowed due to low numbers of participants in some categories. Highest educational attainment was originally recorded as (below grade 12, grade 12, certificate, diploma, and degree) then recoded to below grade 12, grade 12 and tertiary. (South African students matriculate from high school at grade 12 level). Employment status was categorized as employed and unemployed, and race as African and other.

Clinical variables included time since HIV diagnosis, time on ART, and duration of adherence club membership. These were calculated by subtracting the date a patient tested HIV positive, was initiated on ART or date of adherence club initiation from the month of data collection, February 2016. Participants gave their baseline CD4 counts from their adherence club records. Baseline CD4 count (a participant’s CD4 count when first enrolled in the club) was measured in cells/μl and analysed as log CD4 count to correct for positive skewing inherent in CD4 counts. Body mass index (BMI) was calculated and categorized into three groups: underweight (<18.5 kg/m^2^), normal (18.5–24.9 kg/m^2^) and, overweight/obese (>25.0 kg/m^2^). Overweight and obese were combined due to the low numbers of study participants in the individual categories. Comorbidities (diabetes mellitus, cancer, obesity, heart disease or respiratory disease) were coded as present and absent.

The daily ART pill count was a categorical variable of pills ranging from one to three pills. Individual ART regimens were not recorded at the time of data collection. However, this study took place during a transition from combination ART (cART) to fixed-dose combinations (FDC). Hence, the national ART guidelines were used to infer participant ART regimen based on daily pill count [35,36]. The treatment guidelines are summarized and adapted in Table 1.

**Table 1:** ART regimens as per national department of health guidelines.

ART regimen	Daily pill count	Contraindication	Possible ART regimen
Single pill FDC	1	n/a	**(TDF + FTC + EFV)** combination
Double pill FDC	2	EFV	**(TDF + FTC)** combination + NVP
		TDF	**(AZT + 3TC)** combination + EFV
		TDF + EFV	**(AZT + 3TC)** combination + NVP
		TDF + AZT	**(ABC + 3TC)** combination + EFV
		TDF + AZT + EFV	**(ABC + 3TC)** combination + NVP
		Failing TDF based 1^st^ line treatment	**(AZT + 3TC)** combination + LPV/r (2^nd^ line)
Combination ART	3	n/a	TDF + FTC/3TC + EFV

TDF, tenofovir; FTC, emtricitabine; EFV, efavirenz; NVP, nevirapine; AZT, zidovudine; 3TC, lamivudine; ABC, abacavir; LPV/r, lopinavir/ritonavir.

Adapted from National Department of Health. FDC ARV Circular 25052015.pdf.Pretoria; 2015. Available: https://sahivsoc.org/Files/FDC/ARV/Circular/25052015.pdf [cited: 2020 Aug, 15] [35].

Participants on cART took three individual pills daily. Those on a single pill FDC took a single pill containing tenofovir, lamivudine, and efavirenz daily. People living with HIV/AIDS with contraindications to any of the standard drugs in the single tablet FDC regimen such as allergic reactions to TDF, FTC or EFV were prescribed two pills daily: a two-drug FDC pill (lamivudine plus zidovudine, tenofovir, or abacavir) and another pill containing efavirenz or nevirapine. Patients on second-line treatment had similar drug combinations except that efavirenz was replaced with a lopinavir and ritonavir (LPV/r) pill or, in some cases, lamivudine was replaced with emtricitabine [35,36]. Newly diagnosed and pregnant PLWHA were given priority to convert from cART to FDC regimens. PLWHA experiencing adverse reactions to stavudine-based regimens and those with TB and other comorbidities were also prioritized. As a result, some participants were on a single pill TDF + FTC + EFV fixed-dose regimen while others were on a TDF + FTC + EFV multi-pill combination ART regimen [35].

### Statistical analysis

The characteristics of study participants were described using frequencies and proportions for categorical variables and, medians and interquartile ranges for continuous variables that were not normally distributed. The log of baseline CD4 count was normally distributed and therefore summarized as a mean and standard deviation. The Kruskal-Wallis test was used to determine associations between continuous exposure variables and the ordinal ART adherence scores. To test associations between categorical exposure variables and ART adherence scores, Fisher’s exact test was used since some cells in the contingency tables between categorical exposure variables and adherence score had five or less counts.

Associations between the ordinal ART adherence score and the exposure variables were initially assessed in univariable ordinal logistic regression analysis. Exposure variables with odds ratios (ORs) that were significant, at p≤0.1, were included in an initial multivariable model. Further variable selection was then carried out using the stepwise backward elimination process. Age, ART regimen, comorbidities, adherence club membership duration and years on ART demonstrated a significant association with ART adherence both in univariate models and following stepwise regression and were thus entered into a multivariable ordinal logistic regression model. Variables that had p <0.05 in the multivariate model were considered significant. Collinearity between years on ART and adherence club membership duration was investigated by examining correlation coefficients and variance inflation factors. Based on *a priori* knowledge, age [11–13] and then sex [13–17] were assessed to determine whether they improved the model fit using the Wald and Likelihood Ratio (LR) tests. Age was retained, but sex did not improve the final model. Thus, the most parsimonious model to describe ART adherence included age, ART regimen, comorbidities, and club membership duration.

### Ethical considerations

This study was approved by the Human Research Ethics Committee (HREC) (Medical) of the University of the Witwatersrand (approval number: M200105). Permission to use data for this study was granted by CARe. In the primary study, informed consent was obtained from each participant prior to the survey. All data were anonymized by removing identifiers before analysis of the data.

## Results

(Table 2). There were 730 participants in this study and 58.2% (n = 425) of them were females. The median age of the participants was 39 years, with an interquartile range of 20 to 69 years. Most study participants (n = 712, 97.5%) were of African descent and nearly 50% (n = 361) of them left school before completing grade 12 and over half (57.4%, n = 419) of the participants were unemployed. Over half (61.1%) of study participants reported highly adherent behaviour. Half of the study participants (50%, n = 365) had a normal BMI classification.

**Table 2:** Frequency distribution of social, demographic, and clinical characteristics of study participants.

Variable	Frequency (n)	Percentage (%)
**Sex**		
Male	305	41.8
Female	425	58.2
**Age (years)**		
18–30	108	14.8
31–50	558	76.4
>50	64	8.8
**Race**		
African	712	97.5
Other	18	2.5
**Marital status**		
Single	436	59.7
Married/Cohabiting	255	34.9
Divorced/Widowed	39	5.3
**Highest education**		
Below grade 12	434	59.5
Grade 12	273	37.4
Tertiary	23	3.1
**Employment status**		
Employed	311	42.6
Unemployed	419	57.4
**BMI**		
Underweight	14	3.0
Normal	365	77.5
Overweight/obese	92	19.5
Missing	259	35.5
**Comorbidities**		
Yes	79	10.8
No	651	89.2
**ART regimen**		
Single pill	601	82.3
Double pill	40	5.5
cART^a^	89	12.9

^a^cART–combination ART.

(Table 3). The mean log baseline CD4 count of the participants was 6.3 (standard deviation = 0.2). The median time since HIV diagnosis for highly adherent club members was 5.2 years (interquartile range = 3.7–6.8 years), whilst their median time on ART was 3.7 years (interquartile range = 2.6–5.4 years). The median adherence club membership duration for this study sample was 2.4 years (interquartile range = 1.3–4.1 years).

**Table 3:** Frequency distribution of clinical characteristics of study participants.

Variable	Median	IQR
**Log baseline CD4 count**	6.8 (mean)	0.7 (sd)
**Years since HIV diagnosis**	5.2	3.7–6.8
**Years on ART**	3.7	2.6–5.4
**Club membership duration**	3.4	4.1

(Table 4). Sex, age, race, marital status, highest educational attainment, employment status, and presence of comorbidities were all associated with ART adherence score. Female participants had a significantly higher proportion of high adherence scores than males) (p = 0.036). Participants aged between 31 and 50 years were more likely to report high adherence scores than participants aged 30 years and below (p = 0.043). Participants who were married or cohabiting were more likely to report high adherence scores than single participants (p<0.001). A higher proportion of participants who had only participated in education below grade 12 level reported high adherence scores compared to participants who had attained grade 12 level education (p = 0.002). Additionally, there was a greater proportion of participants who were unemployed who had high adherence scores compared to the proportion of employed participants with high adherence scores (p = 0.031). Participants who had no comorbidities were more likely to report high levels of ART adherence than participants with comorbidities (p = 0.004). There was no statistically significant difference in the adherence scores of participants by BMI, ART regimen, or club membership duration.

**Table 4:** Comparison of sociodemographic and clinical factors among study participants by ART adherence score.

Variable	Highly Nonadherent	Nonadherent	Neutral	Adherent	Highly Adherent	P-value
**Frequency (%)**	2 (0.3)	14 (1.9)	27 (3.7)	241 (33.0)	446 (61.1)	
**Sex**						
Male	2 (100.0)	5 (35.7)	17 (63.0)	107 (44.4)	174 (39.0)	0.036
Female	0 (0.0)	9 (64.3)	10 (37.0)	134 (55.6)	272 (61.0)	
**Age (years)**						
18–30	1 (50.0)	3 (21.43)	7 (25.9)	35(14.5)	62 (13.9)	0.043
31–50	1 (50.0)	7 (50.0)	17 (63.0)	182 (75.5)	351 (78.7)	
>50	0 (0.0)	4 (28.6)	3 (11.1)	24 (10.0)	33 (7.40)	
**Race**						
African	437 (98.0)	234 (97.1)	26 (96.3)	14 (100.0)	1 (50.0)	0.075
Other	9 (2.0)	7 (2.9)	1 (3.7)	0 (0.0)	1 (50.0)	
**BMI**						
Underweight	6 (2.2)	8 (4.7)	0 (0.0)	0 (0.0)	0 (0.0)	0.556
Normal	211 (75.6)	136 (79.5)	12 (85.7)	5 (83.3)	1 (100.0)	
Overweight/obese	62 (22.2)	27 (57.8)	2 (14.3)	1 (16.7)	0 (0.0)	
**Marital status**						
Single	0 (0.0)	9 (64.3)	11 (40.7)	134 (55.6)	282 (63.2)	<0.001
Married/cohabiting	1 (50.0)	2 (14.3)	12 (44.4)	102 (42.3)	138 (30.9)	
Divorced/widowed	1 (50.0)	3 (21.4)	4 (14.81)	5 (2.1)	26 (5.8)	
**Highest education**						
Below grade 12	0 (0.0)	9 (64.3)	18 (66.7)	148 (61.4)	259 (58.1)	0.002
Grade 12	0 (0.0)	5 (35.7)	6 (22.2)	90 (37.3)	172 (38.6)	
Above grade 12	2 (100.0)	0 (0.0)	3 (11.1)	3 (1.2)	15 (3.36)	
**Employment status**						0.031
Employed	0 (0.0)	1 (7.1)	10 (37.0)	102 (42.3)	198 (44.4)	
Unemployed	2 (100.0)	13 (92.9)	17 (63.0)	139 (57.7)	248 (55.6)	
**Comorbidity**						
Yes	0 (0.0)	4 (28.6)	8 (29.6)	28 (11.6)	39 (8.7)	0.004
No	2 (100.0)	10 (71.4)	19 (70.4)	213 (88.4)	407 (91.3)	
**ART regimen**						
Single pill	2 (100.0)	12 (85.7)	25 (92.6)	205 (85.1)	357 (80.2)	0.526
Double pill	0 (0.0)	1 (7.1)	0 (0.0)	14 (5.81)	25 (5.6)	
cART^a^	0 (0.0)	1 (7.14)	2 (7.4)	22 (9.1)	63 (14.2)	
**Log baseline CD4 count,** **mean (sd)**	6.3 (0.2)	6.3 (0.2)	6.3 (0.2)	6.2 (0.2)	6.4 (-)	0.097
**Median (IQR)**						
**Years since HIV diagnosis**	5.3 (3.6–6.8)	5.2 (3.7–6.5)	5.3 (3.7–7.7)	5.6 (4.8–7.0)	3.8 (1.6–6.2)	0.091
**Years on ART**	3.8 (2.5–5.5)	3.2 (2.6–4.8)	3.4 (2.7–5.9)	4.3 (2.4–5.5)	3.6 (1.3–5.8)	0.059
**Club membership (years)**	2.9 (1.4–4.3)	1.9 (1.3–3.6)	1.9 (1.2–4.8)	2.7 (1.1–3.9)	2.7 (1.1–4.3)	0.370

^a^cART–combination ART.

(Table 5). Factors significantly positively associated with ART adherence score on univariable analysis in HIV-positive adherence club members were female sex, married and cohabiting marital status, and the cART regimen. ART adherence score was negatively associated with increasing years on ART and adherence club membership duration. Univariable analyses showed that the odds of high adherence scores among female participants are 40% higher than the odds of high adherence scores among male participants (p = 0.036) and the odds of high adherence scores among married and cohabiting participants are 30% lower than the odds of high adherence scores among single participants (p = 0.008). The odds of reporting high adherence scores among participants with comorbidities was 50% lower than the odds of reporting high adherence scores among participants with no comorbidities (p = 0.005). Additionally, the odds of reporting high adherence scores among participants on cART was nearly twice as likely (OR = 1.8 p = 0.022) as reporting high adherence scores amongst participants on a single pill ART regimen. Participants were 10% less likely to report high adherence score with increasing years on ART and participants were also 20% less likely to report high adherence scores with increasing adherence club durations.

**Table 5:** Factors associated with ART adherence amongst ART adherence club members in Ekurhuleni Metropolitan Municipality.

Variable	n (%)	^a^OR (95% CI)	P-value
**Sex**			
Male	305 (41.8)	1	
Female	425 (58.2)	1.4 (1.0–1.9)	0.036
**Age**			* *
18–30	108 (14.8)	1	
31–50	558 (76.4)	1.4 (0.9–2.1)	0.150
> 50	64 (8.8)	0.8 (0.4–1.5)	0.460
**Race**			
African	712 (97.5)	1	
Other	18 (2.5)	0.6 (0.2–1.5)	0.268
**BMI**			
Underweight	14 (3.0)	1	
Normal	365 (77.5)	1.5 (0.6–4.2)	0.394
Overweight & obese	92 (19.5)	2.3 (0.8–6.8)	0.120
**Marital status **			
Single	436 (59.7)	1	
Married/cohabiting	255 (34.9)	0.7 (0.5–0.9)	0.008
Divorced/widowed	39 (5.3)	0.8 (0.4–1.7)	0.601
**Highest education**			
Below grade 12	361 (49.5)	1	
Grade 12	273 (37.4)	1.2 (0.9–1.6)	0.304
Above grade 12	23 (3.2)	0.9 (0.4–2.3)	0.894
**Employment Status**			
Employed	311 (42.6)	1	
Unemployed	419 (57.4)	0.8 (0.6–1.1)	0.112
**Comorbidity**			
No	651 (89.2)	1	
Yes	79 (10.8)	0.5 (0.3–0.8)	0.005
**ART regimen**			
Single pill	601 (82.3)	1	
Double pill	40 (5.5)	1.2 (0.6–2.3)	0.592
cART^b^	89 (12.9)	1.8 (1.1–2.9)	0.022
	**Mean (sd)**		
**Log Baseline CD4 count**	6.8(0.7)	1.0 (1.0–1.0)	0.440
	**Median (IQR)**		
**Years HIV positive**	5.2 (3.7–6.8)	1.0 (0.9–1.0)	0.636
**Years on ART**	3.7 (2.6–5.4)	0.9 (0.8–1.0)	0.013
**Club membership duration (years)**	3.4 (1.3–4.1)	0.8 (0.8–0.9)	<0.001

^a^OR–unadjusted odds ratio.

^b^cART–combination ART.

The presence of comorbidities, ART regimen, years on ART and adherence club membership duration remained statistically significant on multivariable analyses. However, years on ART was removed from the final multivariable model as it displayed collinearity with adherence club membership duration (variance inflation factor (VIF): 16.76; correlation coefficient: 0.9696). Although the association between age and ART adherence score was marginal (p = 0.073) at the 5% significance level, age was retained in the model as it improved the model fit (Likelihood ratio = 7.32, p = 0.026). The inclusion of sex did not improve the model fit of the multivariable model (Wald p-value = 0.4115). Thus, it was not retained in the final model. Therefore, the final model describing factors associated with ART adherence among PLWHA attending ART adherence clubs included age.

(Table 6) The odds of reporting high adherence scores among participants aged between 31 and 50 (AOR = 1.6, 95% CI: 1.0–2.5, p-value = 0.073) years were 60% as likely as the odds of high adherence scores among participants aged between 18 and 30 years, although this association was only marginally significant. After adjusting for age, participants with comorbidities were half as likely to report high ART adherence scores compared to participants without comorbidities (AOR = 0.5, 95% CI: 0.3–0.8, p = 0.005). Participants on combination ART remained nearly twice as likely to report high adherence scores as participants on single pill fixed dose regimens (AOR = 1.8, 95% CI: 1.0–3.2, p = 0.033). There was a significant reduction in the odds of reporting high ART adherence scores for each additional year of adherence club membership. The odds of reporting high adherence scores dropped by 20% for each successive year in an adherence club (AOR = 0.8, 95% CI: 0.8–0.9, p<0.001).

**Table 6:** Factors associated with ART adherence amongst ART adherence club members in Ekurhuleni Metropolitan Municipality in multivariable analysis.

Factor	n (%)	AOR^a^ (95% CI)	P-Value
**Age (years)**			
18–30	108 (14.8)	1	
31–50	558 (76.4)	1.6 (1.0–2.5)	0.073
> 50	64 (8.8)	0.8 (0.4–1.6)	0.583
**Comorbidity**			
No	651 (89.2)	1	
Yes	79 (10.8)	0.5 (0.3–0.8)	0.005
**ART regimen**			
Single pill	601 (82.3)	1	
Double pill	40 (5.5)	1.3 (0.6–3.0)	0.489
cART^b^	89 (12.9)	1.8 (1.0–3.2)	0.033
**Club membership duration (years)**		0.8 (0.8–0.9)	<0.001

^a^AOR–adjusted odds ratio.

^b^cART–combination ART.

## Discussion

This study investigated factors associated with ART adherence among HIV-positive adults attending adherence clubs in Ekurhuleni Metropolitan Municipality. The presence of comorbidities was associated with low ART adherence, the cART regimen was associated with higher ART adherence, and long adherence club membership durations were associated with low ART adherence.

The presence of comorbidities was associated with a decrease in ART adherence, which was consistent with previous studies [16,37]. This association was attributed to pill fatigue as PLWHA with comorbidities undergo simultaneous treatment for HIV and their specific comorbidities [16]. The resulting drug interactions may result in adverse drug interactions that pose a further threat to ART adherence [21,38]. Thus, lifestyle interventions, coping with side effects, and pill burden should be discussed in adherence counselling sessions for the benefit of adherence club members with comorbidities.

Multi pill regimens (cART) were associated with higher levels of adherence to ART than individuals on single-pill (FDC) regimens. This finding was unexpected as previous studies have shown that treatment regimens with higher pill burdens result in nonadherence [17,39]. However, primary healthcare nurses in Limpopo have reported that their patients often abandon ART after experiencing health improvements as they believed that ART had cured them of HIV/AIDS and was not a lifelong treatment [19]. Thus, study participants on a single pill regimen may have experienced positive health outcomes and become nonadherent because they believed that they were cured. Adherence club facilitators, therefore, need to emphasize the need to take ART as a lifelong treatment during counselling sessions, especially among those taking single dose regimens.

The longer an individual was an adherence club member, the less likely they were to adhere to ART. While this factor has not been investigated before, researchers have demonstrated that ART treatment duration has a negative effect on adherence which has mainly been attributed to treatment fatigue [26,40]. A more effective adherence club model could include the regular use of screening tools. For instance, adherence club facilitators could use questionnaires to assess treatment fatigue, which would allow early detection and targeted counselling of adherence club members experiencing treatment fatigue.

Age was marginally associated with ART adherence. Club members aged between 31 and 50 years of age were more likely to demonstrate highly adherent behaviour than those 30 years old and below. This is consistent with findings by Larsen and colleagues who argued that younger PLWHA experience discrimination in healthcare settings which may result in disengagement [13].

### Limitations

Adherence clubs function differently according to varying settings [41] and are run differently from regular clinics [42]. Therefore, results from this study cannot be generalized to ART adherence clubs in other settings or to HIV-positive individuals receiving regular clinical care. Information bias presented a major limitation as the data were not complete, and several variables such as recent CD4 counts, baseline viral load, and recent viral load had to be disregarded from the study as their inclusion would have resulted in a smaller sample size, thereby limiting the power of the study. Recent viral load, for instance had as many as 600/730 (82%) missing observations. Self-reported measures were used for the outcome variable and several exposure variables. Thus, reporting bias could have occurred if participants reported inaccurate details.

## Conclusion

This study has demonstrated associations between long adherence club memberships, single-pill regimens, and comorbidities with an increased likelihood to report low ART adherence among ART adherence club members in Ekurhuleni. These findings provide information that may enable adherence club stakeholders to review and optimize the adherence club model by prioritizing targeted counselling and developing screening tools to aid early detection of treatment fatigue and thereby increase reported ART adherence.

## Supporting information

S1 Dataset (DTA)
